# Are students ready for meaningful use?

**DOI:** 10.3402/meo.v18i0.22495

**Published:** 2013-11-19

**Authors:** Gary S. Ferenchick, David Solomon, Asad Mohmand, Basim Towfiq, Kevin Kavanaugh, Larry Warbasse, James Addison, Frances Chames

**Affiliations:** 1 Division of General Medicine, Department of Medicine, College of Human Medicine, Michigan State University, East Lansing, MI, USA; 2 Division of General Medicine and Office of Medical Education Research and Development, College of Human Medicine, Michigan State University, East Lansing, MI, USA; 3 Virginia Commonwealth University, Cardiology Fellow, Richmond, VA, USA; 4 Department of Medicine, Michigan State University College of Human Medicine, Flint Hurley Campus, Flint, MI, USA; 5 Department of Medicine, Michigan State University College of Human Medicine, Kalamazoo Campus, Kalamazoo, MI, USA; 6 Department of Internal Medicine, Michigan State University College of Human Medicine, Munson Campus, Traverse City, MI, USA; 7 Department of Internal Medicine, Michigan State University College of Human Medicine, Marquette General Campus, Marquette, MI, USA; 8 Department of Medicine, Michigan State University College of Human Medicine Grand Rapids Campus, Grand Rapids, MI, USA

**Keywords:** documentation/methods, electronic health records, professional competence, students, medical, curriculum

## Abstract

**Background:**

The meaningful use (MU) of electronic medical records (EMRs) is being implemented in three stages. Key objectives of stage one include electronic analysis of data entered into structured fields, using decision-support tools (e.g., checking drug–drug interactions [DDI]) and electronic information exchange.

**Objective:**

The authors assessed the performance of medical students on 10 stage-one MU tasks and measured the correlation between students’ MU performance and subsequent end-of-clerkship professionalism assessments and their grades on an end-of-year objective structured clinical examination.

**Participants:**

Two-hundred and twenty-two third-year medical students on the internal medicine (IM) clerkship.

**Design/main measures:**

From July 2010 to February 2012, all students viewed 15 online tutorials covering MU competencies. The authors measured student MU documentation and performance in the chart of a virtual patient using a fully functional training EMR. Specific MU measurements included, adding: a new problem, a new medication, an advanced directive, smoking status, the results of screening tests; and performing a DDI (in which a major interaction was probable), and communicating a plan for this interaction.

**Key results:**

A total of 130 MU errors were identified. Sixty-eight (30.6%) students had at least one error, and 30 (13.5%) had more than one (range 2–6). Of the 130 errors, 90 (69.2%) were errors in structured data entry. Errors occurred in medication dosing and instructions (18%), DDI identification (12%), documenting smoking status (15%), and colonoscopy results (23%). Students with MU errors demonstrated poorer performance on end-of-clerkship professionalism assessments (*r =*−0.112, *p*=0.048) and lower observed structured clinical examination (OSCE) history-taking skills (*r =*−0.165, *p*=0.008) and communication scores (*r*= − 0.173, *p*=0.006).

**Conclusions:**

MU errors among medical students are common and correlate with subsequent poor performance in multiple educational domains. These results indicate that without assessment and feedback, a substantial minority of students may not be ready to progress to more advanced MU tasks.

## Introduction

The Health Information Technology for Economic and Clinical Health (HITECH) act was legislated in 2009. It establishes targets for the meaningful use (MU) of electronic medical records (EMRs). MU is being implemented in three stages over several years with the aim of improving healthcare outcomes (1). Since MU is a cornerstone of EMR initiatives, it is associated with measurable benchmarks specifically related to *how well* EMRs are used.

Key measures of the first stage of the HITECT act include the use of structured fields for data entry that will facilitate the electronic measurement of patient care delivery and outcomes. Most of these key measures represent the ‘low hanging fruit’ of effective EMR use, that is, measures that can be easily implemented, used by practicing physicians and taught to medical students. The requirements of the subsequent two stages of MU implementation are progressively more difficult to implement into practice (1). Examples of stage-one core measures include using structured data fields to record the patients’ problems, their active medication lists, and their smoking status. Other requirements of this stage include the use of decision-support tools (e.g., checking drug–drug interactions [DDI]) and the capability of exchanging clinical information electronically among health systems and providers.

The readiness of medical students for these MU tasks is undetermined. An estimated 64% of medical students are currently using EMRs at academic medical centers in the United States, a rate that is higher than that for physicians in practice (2). However, how effectively students are using EMRs is largely unknown. The Alliance for Clinical Education (ACE) has published a list of expected student competencies related to EMR use along with suggested evaluation strategies. A key recommendation is to limit students’ use of advanced EMR functions until they can demonstrate competence in the ‘documentation of essential elements’ (2).

Although ‘essential elements’ are not explicitly defined in this report, the accurate and reliable implementation of stage-one measures is essential in achieving stage-two (disease management) and stage-three (improved outcomes) goals. We believe the basic skills taught and demonstrated through this exercise are key components of these ‘essential elements’.

The objectives of this study were to assess the performance of medical students on 10 discrete stage-one-specific MU requirements (defined below) and to determine if the students’ MU performance correlated with other measures of educational attainment.

## Methods

### Student EMR MU measures

The College of Human Medicine at Michigan State University (MSU) is a community-based medical school with clinical training in seven communities throughout Michigan. The study took place during the 8-week internal medicine (IM) clerkship between July 2010 and February 2012. During this time, as part of their IM clerkship requirements, all 222 third-year medical students viewed 15 online tutorials related to basic EMR functions including MU competencies. The total time for completion of the 15 tutorials was 71 min. After completing the tutorials, students were required to remotely access our EMR playground to complete 10 MU tasks in the chart of a simulated patient.

**Fig. 1:**
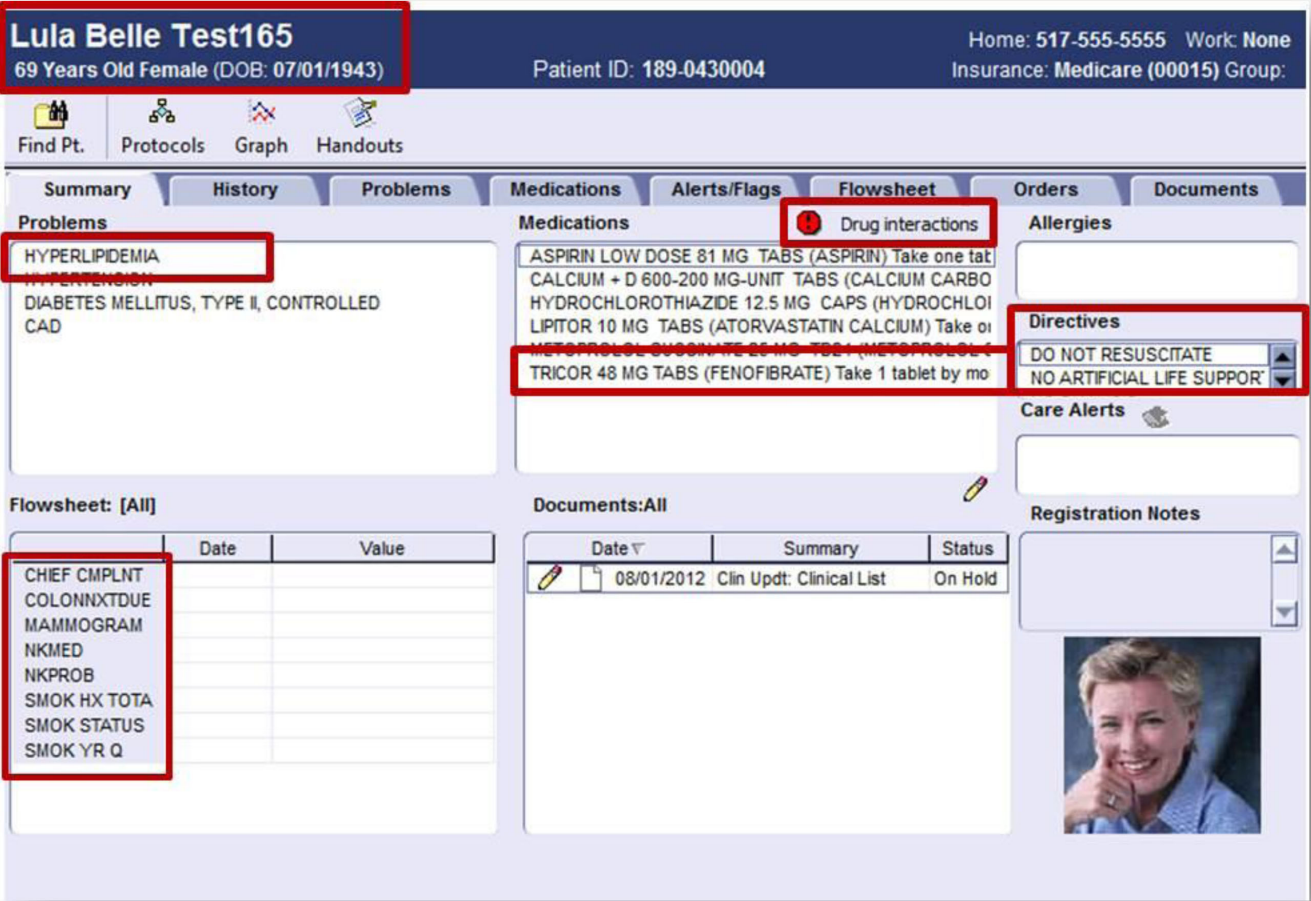
A screenshot of the electronic health record interface for the MU assignment.

**Fig. 2:**
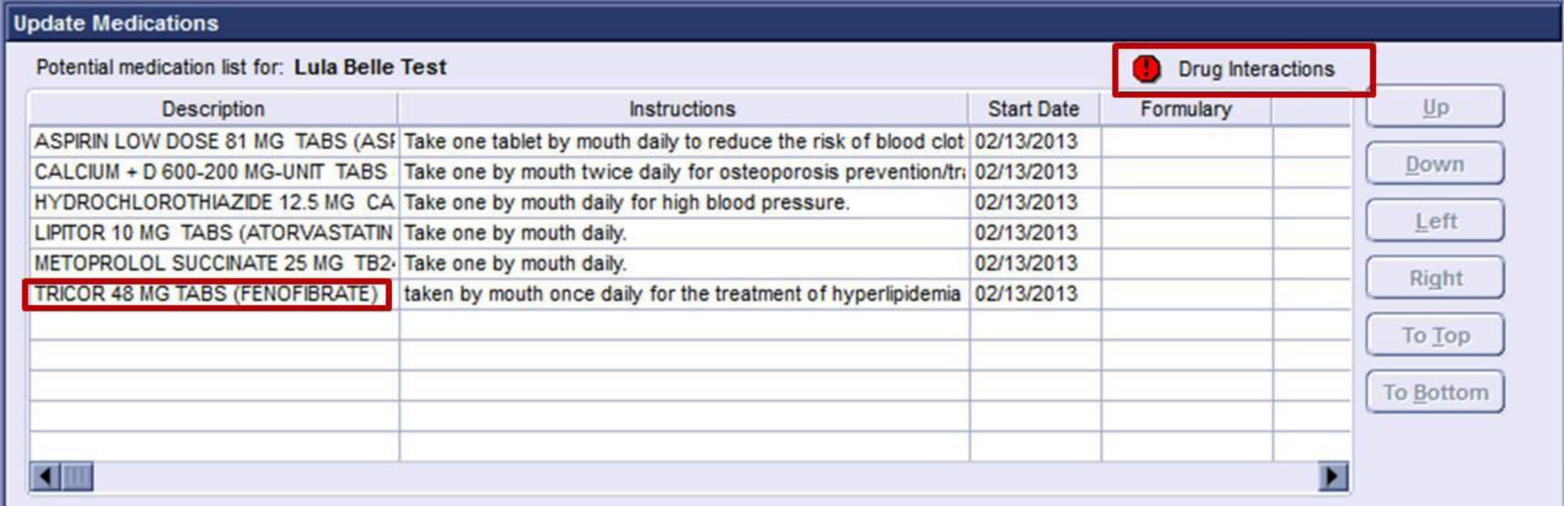
Screenshot of the prompts for student use in identifying the potential drug-drug interaction within the Centricity playground.

**Fig. 3:**
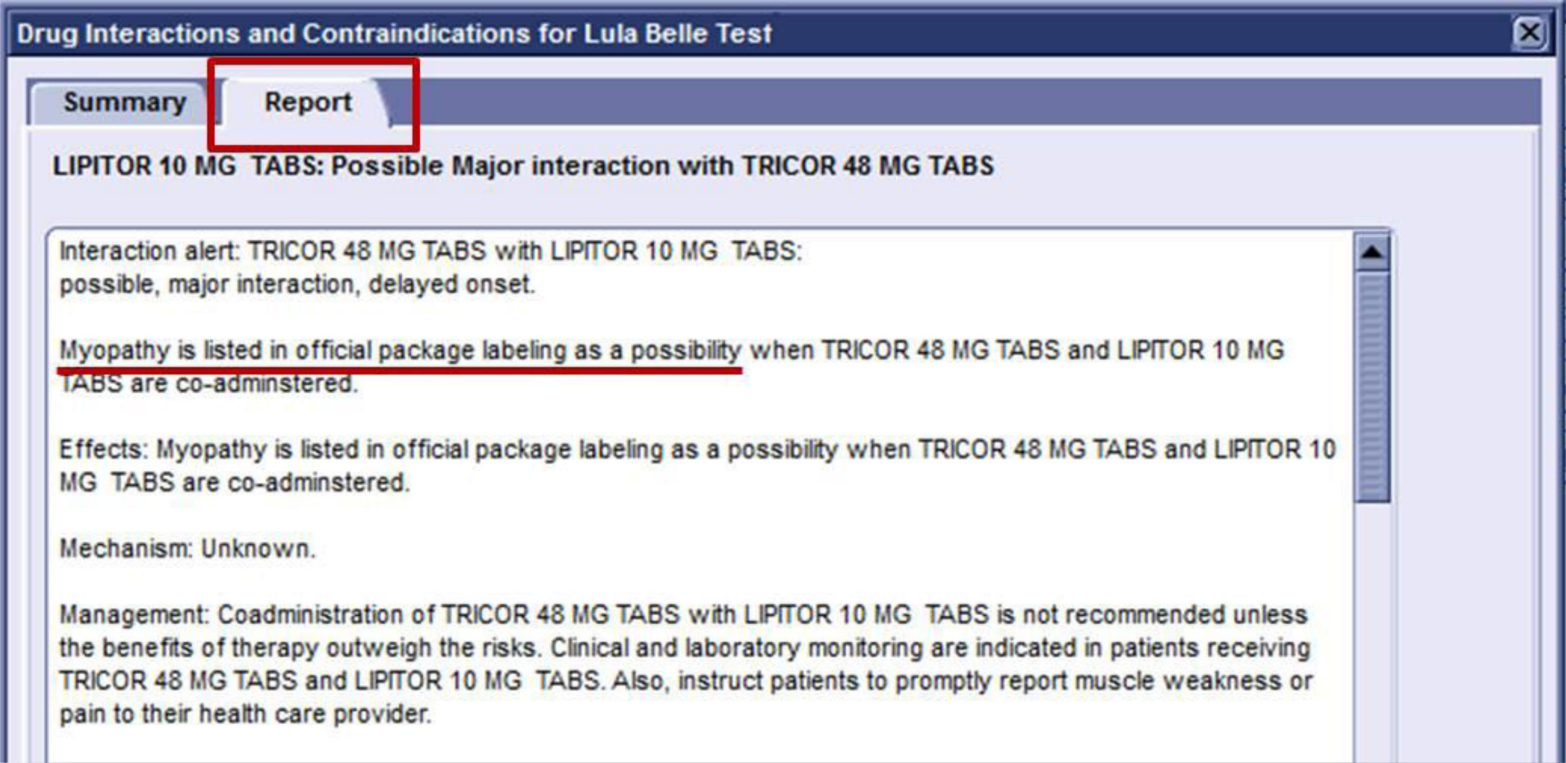
After clicking on the icon, the actual report of the potential interaction was displayed.

**Fig. 4:**
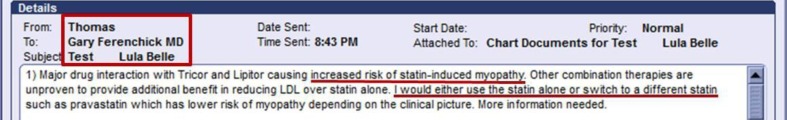
Using the flag function within the electronic health record, the student generated a flag identifying the drug–drug interaction and their recommended management considerations.

Our EMR playground is a fully functional version of Centricity^®^; the clinical EMR for patient care used at MSU but specifically implemented solely for education and development activities and which contains no actual patient data. Simulated virtual patients were created for the purposes of this training and evaluation exercise. After logging in, the students were presented with their personal desktop, onto which the simulated virtual patient's chart had been created. The personal desktop is the interface through which physicians interact with the EMR. Every student had his or her own patient with the exact same information included in the chart. The students were tasked with entering the following information into structured data fields (which collectively represent 10 discrete stage-one MU requirements): a new problem, a new medication, an advanced directive, the patient's smoking status, the results of a recent screening colonoscopy (in which an adenomatous polyp was identified and a 3-year follow-up colonoscopy was recommended) and a recent normal mammogram. Additionally, students were required to perform a DDI in which a moderate interaction resulting in myopathy was possible between the drug they added (fenofibrate) and a drug already on the patients medication list (atorvastatin). Finally, students were tasked with electronically communicating their concern over the DDI and their plan of action to address this interaction. Any plan of action that recommended either not using the fenofibrate at all or continued use with enhanced patient education and/or enhanced surveillance were accepted as reasonable plans of action (Figs. 1–4).

Student's performance for each of the 10 steps on their initial attempt was assessed dichotomously as either being performed correctly or not by a single rater (G. S. F.). Grading was facilitated by the fact all the data entry fields used were structured. The 10 MU competencies we assessed are listed in Table 1.

**Table 1:** List of 10 meaningful use competencies assessed

Students were tasked with adding:
• New problem
• New medication
• Medication dosing and instructions
• Advanced directive
• Results of a recent screening colonoscopy
• Results of a recent screening mammogram
• Smoking status
Students were tasked with performing:
• Electronic communication using a ‘flag’
• Identifying a potential drug–drug interaction
• Communicating a plan of action for the drug–drug interaction

We calculated the following: the total number of MU errors committed, the number of students with at least one MU error, the number students with two or more MU errors, and the frequency and types of MU errors after the students’ first attempt. Students who did not successfully complete each of the 10 steps on their initial attempt were sent specific feedback on their performance, and they were required to complete the record until all 10 MU steps were performed correctly. Students’ performance on their initial attempt at each MU task was utilized for all analyses.

Before entering the fourth year, students must successfully complete a gateway standardized patient (SP) examination which includes a series of SP cases covering a range of common problems and assessing key skills in patient interviewing, physical examination, and counseling/teaching skills. For the purpose of this study, we used overall checklist/rating scores in the areas of communication skills, history taking, and physical examination.

### Predictive value of student MU performance

To define the predictive value of student MU errors for subsequent performances and/or ratings during the third year of medical school, we measured the correlation between MU errors and the students’ National Board of medical Examiners (NBME) subject exam score for IM, their end-of-clerkship professionalism assessments, and their end-of-year gateway OSCE clinical skills scores on history-taking, physical examination, and communication skills. The end-of-clerkship professionalism assessment was the average ‘professionalism score’ received by the student from their clerkship clinical evaluators (e.g., a score of 1 indicated professionalism assessed at below expectations, a score of 2 indicated a professionalism score assessed at expectations, and a score of 3 above expectations).

This project received an ‘exempt’ status by the university IRB.

## Results

Of the 222 students, 68 (30.6%) made one or more MU errors and 30 (13.5%) made two or more errors (range 2–6). The most common type of error was in documenting the results of the patient's recent colonoscopy, which occurred with 23% of the students. The second most common error was in documenting medication dosing and medication use instructions, occurring with 18% of the students. Fifteen percent did not appropriately document the patient's smoking status, and 12–14% either did not find the DDI or did not develop a reasonable plan of action for managing it. Other errors and their frequency of occurrence are noted in Table 2.

**Table 2:** Number and types of meaningful use errors committed by students

	*N*	%
Students with MU errors		
Total number of students	222	
Students with at least one MU error	68	30.6
Students with two or more MU error	30	13.5
Types of MU errors		
Preventive care documentation	30	23
Medication dosing and/or instructions	24	18
Documenting smoking status	20	15
Reasonable plan for DDI	18	14
Identifying DDI	16	12
Adding problem from structured problem list	8	6
Using flag to communicate findings	6	5
Adding a drug from a structured data field	4	3
Adding advanced directive from structured data field	4	3
Total number of MU errors	130	

DDI, drug–drug interaction; MU, meaningful use.

We observed a low but statistically significant inverse Pearson product moment correlation between the number of MU errors made by the students and their subsequent assessments on professionalism and end-of-year OSCE scores for communication and history taking (Table 3).

**Table 3:** Correlation between MU performance and subsequent student performance

Correlation between MU errors and:	*R*	Sig (one-tailed)
NBME score	−0.081	0.117
Professionalism assessment	−0.112	0.048*
OSCE communication score	−0.173	0.006*
OSCE history-taking score	−0.165	0.008*
OSCE physical exam score	−0.087	0.103

MU, meaningful use.

* *p*<0.05.

## Discussion

Although a vast majority of medical students are currently using EMRs at academic medical centers, how effectively students are using them is largely unknown. Specifically, the readiness of medical students to capably accomplish tasks related to MU is undetermined.

A recent survey suggested that clerkship directors in the United States believe that students learn how to use EMRs relatively quickly (2). However, data for this contention are lacking. Previous studies have documented students’ underperformance related to EMR use in spite of EMR-specific training. For example, using student self-report as the outcome measure, Rouf et al. reported that most students underutilized specific EMR features, such as prompts for medication interactions and preventive services (3). Yudkowsky et al. reported that 94% of 197 students accessing an EMR during a simulated challenge failed to retrieve critical information embedded within the EMR (i.e., history of a previous myocardial infarction and thrombocytopenia) related to the SP's chief complaint (4). There was no apparent assessment of students’ EMR competencies prior to actual EMR use in either of these studies.

Simply equating the use of technology with success is a misleading notion. Many stage-one tasks of the HITECH act are those that are essential to creating any medical record. However, a key difference between paper and electronic charts is that the entry of basic patient-specific data occurs using structured data fields in the latter, the ultimate use of which will be the electronic measurement of patient care outcomes. A surprising finding in our study is that a substantial minority of students commits MU errors in ‘low hanging fruit’ tasks, in spite of successfully completing on-line training tutorials. Many of these tasks are relatively straightforward, such as the effective use of pull-down menus or checkboxes for data entry. This study strongly reinforces the ACE recommendations to not only teach but to *assess* basic EMR competencies before students are allowed to use advance EMR functions.

This is also the first study to establish a significant association between performance outcomes related to EMR use and other evaluated educational domains. The reasons for this association are not clear but may relate to global academic performance deficiencies, as suggested by the association between subpar EMR performance and poor performance on multiple other educational outcomes. This finding may point to potential problems with following directions and attention to detail, a finding reinforced by the inverse correlation of MU errors with OSCE history-taking scores. Additionally, this finding may be associated with attitude or behavioral issues as suggested by the inverse correlation of MU errors with professionalism scores. Regardless, we did not find an inverse correlation with MU errors and end-of-clerkship subject examination scores, suggesting that knowledge *per se* was not a predictor of poor MU performance.

Another possible explanation for this association is the concept of conscientiousness as an aspect of professionalism. We believe errors in completing these relatively straightforward EMR tasks may reflect students’ lack of attention to detail or lack of conscientiousness. A number of researchers have developed scales assessing conscientiousness as measured by completing basic required tasks. Examples include course evaluations, submission of immunization records, and attendance in required course sessions. Chaytor et al. demonstrated these behaviors are fairly stable year to year and a pair of studies (McLarchlan et al. and Kelly et al.) found similar measures are consistent with faculty ratings and as in this study, performance on a clinical skills examination (5–7). Lievens et al. using a self-report personality measures found conscientiousness to be moderately related to general performance on examinations in a large cohort of Flemish medical students (8). Finally, Stern et al. found similar measures, such as completion of immunization records and failing to complete evaluations, was predictive of student referral for academic review of potentially serious infractions during clinical training (9). While we presently lack data to test this hypothesis, we feel errors in completing rudimentary tasks in using the EMR may be symptomatic of a of lack of conscientiousness that has been identified by other researchers as a stable trait that may result in future lapses of professionalism in the students’ medical education and professional career.

This generalizability of the findings of this study is limited by the nature of the EMR assignment and the fact that this was a single, institution study. For example, if students were presented with different EMR tasks would their performance results be the same. Second, the nature of our educational methods (i.e., videotaped tutorials) and our specific EMR platform (i.e., Centricity) may have influenced our results. Third, although statistically significant, our correlations were small (between 0.11 and 0.16), suggesting many other potential influences on student performance. Fourth, there is no way in this study to measure student attitudes toward this assignment, it is possible that the cohort of poorly performing students did not take the EMR assignment seriously, knowing that the patient was ‘virtual’ and not real.

Although touted as a means to decrease errors and improve patient safety, improved outcomes do not automatically follow EMR use. The mere presence of an EMR will not improve practice quality and will not necessarily make the educational experience better or more efficient. As reflected in the ACE recommendations, EMR use adds another dimension to the responsibilities of educators, that is, to assure that graduating students have the necessary skills to safely use EMRs as they gain more independence in the care of patients.
